# Prophylactic antibiotic use in pediatric patients undergoing urinary tract catheterization: a survey of members of the Society for Pediatric Urology

**DOI:** 10.1186/s12894-017-0268-5

**Published:** 2017-09-06

**Authors:** Alexander P. Glaser, Ilina Rosoklija, Emilie K. Johnson, Elizabeth B. Yerkes

**Affiliations:** Department of Surgery, Division of Urology, Ann and Robert H. Lurie Children’s Hospital of Chicago, 225 E. Chicago Ave, Chicago, IL 60611 USA

**Keywords:** Urinary tract infection, Catheter-related infection, Antibiotic prophylaxis, Health care survey, Pediatrics

## Abstract

**Background:**

Current organizational guidelines regarding use of antibiotics during urinary tract catheterization are based on limited evidence and are not directly applicable to the pediatric urology population. We seek to improve understanding of this population by first evaluating current practices. This study aims to investigate practice patterns and attitudes of pediatric urologists regarding the use of antibiotics in the setting of urinary tract catheterization.

**Methods:**

An online survey was sent to members of the Society for Pediatric Urology. Questionnaire sections included demographics, general questions about antibiotic use with catheterization, and specific clinical scenarios. Descriptive statistics were used, and chi-square analysis was performed to examine associations between demographics and specific responses.

**Results:**

Of 448 pediatric urologists surveyed, 154 (34%) responded to the survey. A majority of surveyed urologists (78%) prescribe daily prophylactic antibiotics with a hypospadias stent in place, but extensive variation in use of antibiotics was reported with other catheters and tubes. Extensive variation in practice patterns was also reported for three case scenarios regarding antibiotic prophylaxis with catheterization. Urologists > 50 years of age and fellowship-trained urologists were more likely to prescribe antibiotics for hypospadias stents (*p* = 0.02, *p* = 0.03), but no other significant associations between demographic characteristics and antibiotic use were found.

**Conclusions:**

There is substantial variation in practice patterns among surveyed pediatric urologists regarding prophylactic antibiotic use with urinary catheterization. This variation, combined with a lack of objective data and increasing pressure to decrease infectious complications and combat antibiotic resistance, highlights the need for development of management guidelines for this unique population.

**Electronic supplementary material:**

The online version of this article (10.1186/s12894-017-0268-5) contains supplementary material, which is available to authorized users.

This manuscript was presented at the American Urological Association annual meeting in 2016 (MP55–15), and is published in abstract form [1].

## Background

Catheter-associated urinary tract infections (CAUTIs) are the most common nosocomial infection in the United States and lead to increased cost of care as well as patient length-of-stay, morbidity, and mortality [2]. Recent national measures in the United States have sought to prevent and decrease the frequency of CAUTIs in the general population [3]. Furthermore, there has been increasing pressure from payers, including the Centers for Medicare & Medicaid Services, to decrease infectious complications by imposing financial penalties on hospitals that perform poorly with regard to hospital-acquired conditions.

Alongside pressure to decrease infectious complications there has been an increasing focus on the problems caused by antibiotic resistance [4]. According to the U.S. Center for Disease Control and Prevention, each year over 2 million illnesses and over 20,000 deaths are directly attributable to antibiotic resistance [4]. Problematic resistance patterns have forced urologists to use broader-spectrum antibiotics on a routine basis [5]. Yet despite growing resistance patterns, antibiotic drug development has stymied and few new drugs are being developed [6–8]. Antibiotic stewardship has been proposed as a solution to promote use of optimal antibiotic regimens; however, due to a lack of evidence in the pediatric urology population, further research is needed to define appropriate and inappropriate antibiotic use [9, 10]. Current guidelines from the American Urological Association (AUA) and the European Association of Urology (EAU) recommend antibiotic prophylaxis with urinary tract catheter removal if bacteriuria and other risk factors (such as older age, smoking status, deficient nutritional status, immunosuppression, diabetes mellitus, and prolonged hospitalization) are present; however, these are based on limited evidence and are not directly applicable to the pediatric urology population [11–13]. Therefore, we sought to improve understanding of this unique population by first describing and measuring current practices. This study aimed to investigate current practice patterns and attitudes of pediatric urologists regarding the use of prophylactic antibiotics in patients undergoing urinary tract catheterization.

## Methods

### Survey and data collection

A 20-item online questionnaire regarding the use of prophylactic antibiotics with urinary catheterization was sent to 315 active and affiliate members of the Society for Pediatric Urology (SPU). The original request was sent via email in August, 2015 and two follow-up reminders were sent in September, 2015. Questionnaire sections included: (1) Demographics, (2) General questions about antibiotic use with urinary catheterization, and (3) Specific clinical scenarios (see Additional file 1). Study data were collected and managed using REDCap (Research Electronic Data Capture, http://project-redcap.org) electronic data capture tools hosted at Northwestern University [14]. This study received institutional review board approval (IRB #2015–462).

### Statistical analysis

Descriptive statistics were used, and chi-square analysis was performed to examine associations between demographics and specific responses. Statistical comparisons were 2-sided with a type I error probability set at 0.05. Analysis was performed using SPSS Statistics (IBM, version 22).

## Results

### Respondent demographics

Of 448 members of the SPU surveyed, 154 (34%) responded to the survey (Table 1). SPU members from all AUA sections responded, ranging from 18% of all SPU members located in the New England section to 42% of all SPU members located in the North Central section. There was no statistically significant difference in response rates between sections (*p* = 0.28). The majority of respondents (91%) were fellowship-trained in pediatric urology or were currently in fellowship. Sixty-six percent of respondents practiced in an academic setting, while the remainder practiced in private practice or as a hospital employee.

**Table 1:** Respondent Demographics

Question	Responses, n (%)
Age (years)	
31–40	35 (23%)
41–50	42 (27%)
51–60	51 (33%)
> 60	25 (16%)
Gender	
Male	116 (75%)
Female	34 (22%)
Other	2 (1.3%)
Fellowship Trained	
Currently in fellowship	6 (4%)
Yes	134 (87%)
No	11 (7%)
Years in practice	
Currently in fellowship	6 (4%)
0–5	29 (19%)
6–10	23 (15%)
11–15	23 (15%)
16–20	21 (14%)
> 20	51 (33%)
Number of pediatric urologists in practice	
1–2	57 (37%)
3–4	51 (33%)
5–6	28 (18%)
7–10	15 (9.7%)
> 10	3 (1.9%)
Practice Setting	
Academic affiliation	102 (66%)
Hospital employee	17 (11%)
Private Practice	34 (22%)
Other	1 (0.6%)
AUA Section	
Mid Atlantic	16 (10%)
New England	6 (4%)
New York	9 (6%)
North Central	38 (25%)
Northeastern	9 (6%)
South Central	20 (13%)
Southeastern	24 (16%)
Western	29 (19%)
From another geographic location	3 (2%)

### Use of prophylactic antibiotics with urinary tract catheterization

**Fig. 1:**
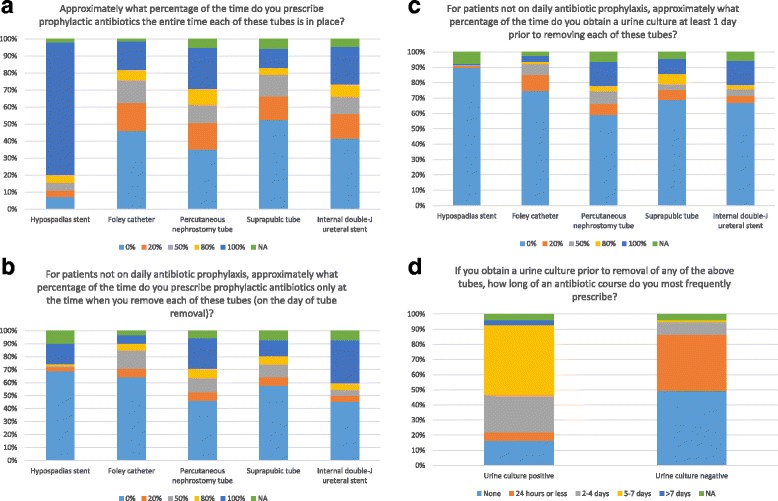
**a** Use of prophylactic antibiotics with urinary tract catheterization the entire time a tube is in place **b** Use of prophylactic antibiotics only at the time of tube removal. **c** Use of urine cultures prior to tube removal **d** Use of culture data and length of antibiotic duration

The majority of respondents (78%) prescribe prophylactic antibiotics the entire time a hypospadias stent is in place (Fig. 1a). However, extensive variation in prescribing patterns was seen for prophylactic antibiotics with use of a Foley catheter, percutaneous nephrostomy tube (PCN), suprapubic tube (SPT), and internal double-J ureteral stent, with 30–50% of respondents prescribing no antibiotics for these tubes, and the remainder prescribing prophylactic antibiotics at least some of the time. The majority of respondents do not prescribe a dose of prophylactic antibiotics at the time of tube removal, with the exception of removal of a ureteral stent (Fig. 1b).

### Use of urine cultures and culture data with urinary tract catheterization

The majority of respondents do not obtain urine cultures prior to removal of a hypospadias stent (90%), Foley catheter (75%), PCN (59%), SPT (69%), or internal double-J ureteral stent (67%) (Fig. 1c). If a urine culture is positive, 46% of respondents prescribe a 5–7 day course of antibiotics, while 25% prescribe 2–4 days of antibiotics, 6% prescribe 24 h of antibiotics, and 16% prescribe no antibiotics (Fig. 1d). If a urine culture is negative, 37% still prescribe 24 h or less of antibiotics, while 49% prescribe no antibiotics.

### Use of prophylactic antibiotics with simple outpatient procedures

**Fig. 2:**
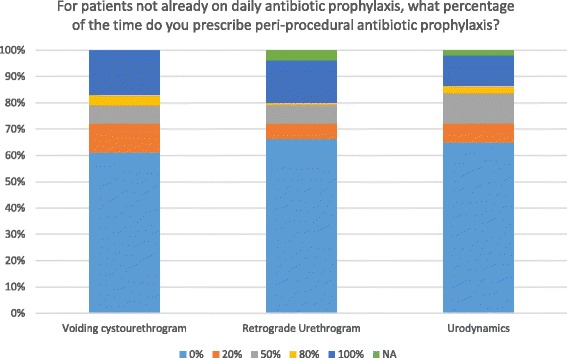
Use of prophylactic antibiotics with outpatient procedures

Sixty-one percent of respondents reported prescribing no prophylactic antibiotics for patients undergoing a voiding cystourethrogram, while the remainder (39%) prescribe prophylactic antibiotics at least some of the time (Fig. 2). Similar variation is seen for retrograde urethrogram and urodynamic studies.

### Use of antibiotics in clinical case scenarios

**Fig. 3:**
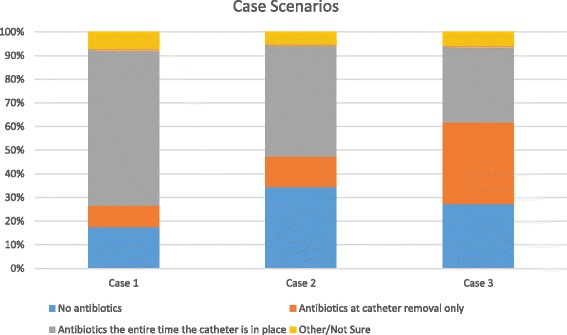
Case Scenarios. Case 1: An 8 year-old male with a history of previous hypospadias repair and short urethral stricture undergoes cystoscopy with direct visual internal urethrotomy and you plan to leave a Foley catheter in for 3 days. There is no evidence of current infection. You would administer: Case 2**:** A 12 year old female with a history of spina bifida is being discharged from the hospital after bladder augmentation with creation of a catheterizable channel. You plan to see the patient in the office in 2 weeks for removal of the catheter and self-catheterization teaching. There is no evidence of current infection. You would administer: Case 3: A 2 year old female has a percutaneous nephrostomy tube placed for acutely symptomatic ureteropelvic junction obstruction. There is no evidence of current infection. You plan to leave the percutaneous nephrostomy tube in for 2 weeks. You would administer

Substantial variation in practice patterns was reported for three case scenarios with indwelling catheters and no evidence of infection (Fig. 3). Full case descriptions are detailed in Fig. 3.

For an 8-year old male undergoing a direct visual internal urethrotomy and a subsequent indwelling catheter for 3 days, 66% of respondents prescribe antibiotics the entire time the catheter is in place, while 18% would prescribe antibiotics only at catheter removal, and 9% would prescribe no antibiotics.

For a 12-year old female being discharged from the hospital with a newly created continent catheterizable channel and an indwelling catheter through the channel for 2 weeks, 47% of respondents would prescribe antibiotics the entire time the catheter is in place, while 13% would prescribe antibiotics only at catheter removal, and 34% would prescribe no antibiotics.

Finally, for a 2-year old female with ureteropelvic junction obstruction and a PCN for 2 weeks, 32% of respondents prescribe antibiotics the entire time the PCN is in place, while 34% would prescribe antibiotics only at PCN removal, and 27% would prescribe no antibiotics.

### Personal experience with infectious complications

Twenty-five percent of respondents reported having a patient with a serious complication (requiring an intensive care unit or an invasive procedure) or death related to a CAUTI. Thirty-one percent of these respondents reporting changing their practice based on this event, while 59% reported not changing their practice based on this event, and 10% reported being unsure if their practice changed based on this event.

### Associations

Urologists > 50 years of age and fellowship-trained urologists were more likely to prescribe antibiotics > 50% of the time for hypospadias stents (95% vs. 82%, *p* = 0.02; 95% vs. 79%, *p* = 0.03). Respondents who reported changing practice patterns based on a serious complication or death related to CAUTI were more likely to prescribe antibiotics > 50% of the time with a ureteral double-J stent and for the entire time the catheter is in place for Case Scenario #2 (54% vs. 16%, *p* = 0.04; 60% vs 16%, *p* = 0.02). There was also a possible association noted for respondents who reported changing practice patterns based on an infectious complication to prescribe antibiotics > 50% of the time with a SPC, but this was not statistically significant (42% vs. 8%; *p* = 0.06). There was no difference in prescribing pattern based on gender, size of practice, practice setting, or AUA section.

## Discussion

This survey of SPU members indicates that substantial variation exists in the use of prophylactic antibiotics in pediatric urology patients undergoing short-term urinary catheterization. Almost 80% of all survey respondents always prescribe with a hypospadias stent, but this was the only queried scenario with a clear consensus; extensive variation was seen in the remainder of scenarios and questions. For example, in Case Scenario #3, respondents were split with approximately 1/3 prescribing no antibiotics, 1/3 prescribing antibiotics at catheter removal only, and 1/3 prescribing antibiotics the entire time the catheter is in place. This variation speaks to the complexity of these patients, but also to the lack of evidence guiding antibiotic use.

Interestingly, in our study, respondents who reported changing their practice patterns based on an infectious complication were significantly more likely to prescribe antibiotics for internal double-J stents and for a newly created catheterizable channel (*p* = 0.04, *p* = 0.02). In addition, there was a possible association between respondents who changed their practice based on an infectious complication and more antibiotic use in patients with a SPC (*p* = 0.06). This suggests that the lack of evidence regarding antibiotic use in these populations may allow the availability bias of a prior adverse event to drive decision making.

The lack of consensus in antibiotic prescribing patterns in our study is similar to the survey of Wazait et al., who found that 60% of 237 healthcare professionals prescribe prophylactic antibiotics with urinary catheter removal for adult patients, while 40% did not use antibiotics [15]. Variation has also been reported for preoperative surgical antibiotic prophylaxis in a wide range of endoscopic, laparoscopic, and open surgical procedures in both the adult and pediatric populations [16–18]. While these studies examined preoperative surgical prophylaxis instead of antibiotic use with catheters, the similar variation in antibiotic use corroborates our findings.

In our study, urologists > 50 years of age and fellowship-trained urologists were more likely to prescribe antibiotics at least 50% of the time for hypospadias stents. Several studies have investigated the topic of antibiotic use with hypospadias stents, which may be why this was the only scenario with a clear consensus in our study, and especially for older urologists and fellowship-trained urologists, who may be more familiar with the literature. In 2004, Meir et al. reported a decreased risk of complicated UTI and a non-significant suggestion of a decreased rate of urethrocutanous fistula formation with antibiotic use while a hypospadias stent is in place [19]. However, this result has not been replicated in other studies and the role of prophylactic antibiotics in hypospadias stents remains controversial [20, 21]. A multi-institutional randomized controlled trial is currently ongoing to investigate the role of antibiotics in this setting (PROPHY, clinicaltrials.gov identifier NCT02096159).

The relative lack of evidence guiding antibiotic use with catheterization in the pediatric population may explain the practice variation reported in our study. While there is some evidence that antibiotic use at the time of catheter removal can decrease symptomatic UTIs in adults [22, 23], these results are not consistently reported [24] and antibiotic use in this setting remains controversial [25]. A recent Cochrane review of adult patients found limited evidence that prophylactic antibiotics reduce the incidence of bacteriuria, with even less evidence that this reduces febrile morbidity in those receiving antibiotic prophylaxis [26]. There is also limited evidence that antibiotics reduce bacteriuria for urodynamic studies in adults, but not enough evidence to suggest that antibiotics reduce symptomatic UTIs [27–29].

Variation in antibiotic use in our study may also be explained by a lack of specific management guidelines in this area. Organizational guidelines from the AUA, EAU, and others have limited applicability to the pediatric urology population, although they include the option to give antibiotics in complex scenarios. The AUA Best Practice Policy Statement on Urologic Surgery Antimicrobial Prophylaxis recommends antibiotic prophylaxis for removal of external urinary catheters and for urodynamics if risk factors such as urinary anatomic abnormalities, immunodeficiency, externalized catheters, colonized exogenous or endogenous material, and prolonged hospitalization are present [11]. Many pediatric urology patients will have one or more of these risk factors, yet in our survey many prescribers do not routinely give antibiotics in scenarios that include these risk factors, such as an externalized catheter. The AUA statement suggests that, for patients with risk factors, antimicrobial use at the time of catheter removal may be therapeutic in the setting of prolonged catheterization following a procedure, but the statement does not make a recommendation whether empiric antibiotics or culture-directed therapy is preferable in this setting. Evidence for this is primarily based on adult and post-prostatectomy literature and again may have limited applicability to the pediatric urology population [22, 30]. The EAU guideline on urological infections similarly states that when continuous drainage is in place after surgery, prolonged perioperative antibiotic prophylaxis is not routinely recommended; however, asymptomatic bacteriuria may be treated after removal of the catheter [12]. Finally, the United States Healthcare Infection Control Practices Advisory Committee, a division of the Centers for Disease Control and Prevention, also does not recommend routine antibiotics with short- and long-term catheterization, but does make a specific exception for patients with bacteriuria upon catheter removal following urologic surgery [3].

Although current guidelines include the option to give antibiotics in complex patients, there remains little guidance on which of these patients will actually benefit from antibiotic use. Our study shows that these patients are managed much differently by different practitioners – some with antibiotics, and some without. The risks of overuse of antibiotics are well-documented and include cost, unnecessary drug exposure, potential for allergic reactions, and antimicrobial resistance [2, 4]. Clinician-driven antibiotic stewardship has been proposed as the answer to reducing unnecessary antibiotic use [5, 9]. However, at this time, more directed evidence is needed to guide antibiotic use in pediatric urology. In this study, we approached this broad problem by first determining practice patterns. Next steps to establish effective stewardship include defining appropriate and inappropriate antibiotic use in this population and determining which patients will benefit the most.

Several limitations of this study should be acknowledged. First, only one-third of SPU members responded to our survey; however, this response rate is similar to other previous SPU surveys, and all sections of the AUA were represented in our sample [17, 31]. Also, it is unknown to us how many practicing pediatric urologists are not members of the SPU and were therefore not sent questionnaires; however, responders to our survey included academic, hospital-employed, and private-practice pediatric urologists. Antimicrobial resistance patterns may be different in different demographic areas, but we did not see a difference in prescribing patterns based on AUA section. Associations reported in this study are limited due to the lack of consensus for the majority of queried scenarios. Questions regarding specific antimicrobial agents were not asked. Also, in our study we cannot determine to what extent the reported responses correspond to actual clinical practice. However, we attempted to simulate the clinical environment in our survey by both asking general questions and using clinical scenarios. Finally, in this study we do not provide any information about what the best antimicrobial practice actually is, as this was not the study intent. Instead, this investigation was designed to define and measure current practice patterns as a first step in improving antimicrobial use for pediatric urology patients whose treatments require catheter use.

## Conclusions

Our results indicate there is substantial variation in practice patterns among surveyed pediatric urologists regarding prophylactic antibiotic use with urinary catheterization and minor lower urinary tract procedures such as urodynamics, retrograde urethrogram, and voiding cystourethrogram. This lack of consensus in current management of these complex patients highlights the need for further research in this area and for the development of management guidelines for this unique population.

## Additional file

Additional file 1: Antibiotic use in Pediatric Urology. This supplemental material contains the full survey sent out to members of the SPU. (DOCX 23 kb)

## Abbreviations

AUA: American Urological Association
CAUTI: Catheter-associated urinary tract infection
EAU: European Association of Urology
PCN: Percutaneous nephrostomy tube
SPT: Suprapubic tube
SPU: Society for pediatric urology
UTI: Urinary tract infection
