# Supplementary material for: Prophylactic antibiotic use in pediatric patients undergoing urinary tract catheterization: a survey of members of the Society for Pediatric Urology
Source: BMC Urol. 2017 Sep 6;17:76. doi: 10.1186/s12894-017-0268-5 (PMC5586033; doi:10.1186/s12894-017-0268-5)
Supplement: Additional file 1: — Antibiotic use in Pediatric Urology. This supplemental material contains the full survey sent out to members of the SPU. (DOCX 23 kb) [file 12894_2017_268_MOESM1_ESM.docx]

Antibiotic Use in Pediatric Urology

**Section I. Antibiotic Use**

**Instructions:** Please select one response related to your general practice for patients who have temporary tubes placed into the urinary tract. For these questions, assume the tube has been or will be in place for ≥3 days and that the patient does not have an active infection at the time of tube placement.

1. What percentage of the time do you prescribe prophylactic antibiotics for the entire time each of these tubes is in place?

|  | (1) 0% | (2) 20% | (3) 50% | (4) 80% | (5) 100% | N/A – I don’t use this tube |
| --- | --- | --- | --- | --- | --- | --- |
| Hypospadias stent |  |  |  |  |  |  |
| Foley catheter |  |  |  |  |  |  |
| Percutaneous nephrostomy tube |  |  |  |  |  |  |
| Suprapubic tube |  |  |  |  |  |  |
| Internal double-J ureteral stent |  |  |  |  |  |  |

1. For patients not on daily antibiotic prophylaxis, what percentage of the time do you obtain a urine culture at least 1 day prior to removing each of these tubes?

|  | (1) 0% | (2) 20% | (3) 50% | (4) 80% | (5) 100% | N/A – I don’t use this tube |
| --- | --- | --- | --- | --- | --- | --- |
| Hypospadias stent |  |  |  |  |  |  |
| Foley catheter |  |  |  |  |  |  |
| Percutaneous nephrostomy tube |  |  |  |  |  |  |
| Suprapubic tube |  |  |  |  |  |  |
| Internal double-J ureteral stent |  |  |  |  |  |  |

1. For patients not on daily antibiotic prophylaxis, what percentage of the time do you prescribe prophylactic antibiotics only at the time when you remove each of these tubes (on the day of tube removal)?

|  | (1) 0% | (2) 20% | (3) 50% | (4) 80% | (5) 100% | N/A – I don’t use this tube |
| --- | --- | --- | --- | --- | --- | --- |
| Hypospadias stent |  |  |  |  |  |  |
| Foley catheter |  |  |  |  |  |  |
| Percutaneous nephrostomy tube |  |  |  |  |  |  |
| Suprapubic tube |  |  |  |  |  |  |
| Internal double-J ureteral stent |  |  |  |  |  |  |

1. If you obtain a urine culture prior to tube removal and it is positive, how long of an antibiotic course do you most frequently prescribe?
   1. 1 dose only
   2. 24 hours
   3. 2-4 days
   4. 5-7 days
   5. More than 7 days
   6. N/A – I would not prescribe antibiotics
2. If you obtain a urine culture prior to tube removal and it is negative, how long of an antibiotic course do you most frequently prescribe?
   1. 1 dose only
   2. 24 hours
   3. 2-4 days
   4. 5-7 days
   5. More than 7 days
   6. N/A – I would not prescribe antibiotics

**Instructions:** Please select one response related to your general practice for patients who are having outpatient procedures that require catheterization.

1. For patients not already on daily antibiotic prophylaxis, what percentage of the time do you prescribe peri-procedural antibiotic prophylaxis?

|  | (1) 0% | (2) 20% | (3) 50% | (4) 80% | (5) 100% | N/A – I don’t use this procedure |
| --- | --- | --- | --- | --- | --- | --- |
| Voiding cystourethrogram or radionuclide cystogram |  |  |  |  |  |  |
| Retrograde urethrogram |  |  |  |  |  |  |
| Cystometrogram urodynamic testing |  |  |  |  |  |  |

**Section II. Clinical Scenarios**

The next few questions are case scenarios to get a sense for how you would manage each of these patients with respect to their prophylactic antibiotics.

1. Case 1. An 8 year old male with a history of previous hypospadias repair and a short urethral stricture undergoes cystoscopy with direct visual internal urethrotomy and you plan to leave a Foley catheter in for 3 days. You would administer:
   1. Antibiotics the entire time the catheter is in place
   2. Antibiotics at catheter removal
   3. No antibiotics
   4. Other antibiotic regimen (specify)________________________
   5. Not sure
2. Case 2. A 12 year old female with a history of spina bifida is being discharged from the hospital after bladder augmentation with creation of a catheterizable channel. You plan to see the patient in the office in 2 weeks for removal of the catheter and self-catheterization teaching. You would administer:
   1. Antibiotics the entire time the catheter is in place
   2. Antibiotics at catheter removal
   3. No antibiotics
   4. Other antibiotic regimen (specify)________________________
   5. Not sure
3. Case 3. A 2 year old female has a percutaneous nephrostomy tube placed for acutely symptomatic ureteropelvic junction obstruction. There is no evidence of infection. You plan to leave the percutaneous nephrostomy tube in for 2 weeks. You would administer:
   1. Antibiotics the entire time the nephrostomy tube is in place
   2. Antibiotics at nephrostomy tube removal
   3. No antibiotics
   4. Other antibiotic regimen (specify)________________________
   5. Not sure

**Section III. Personal Information**

In this last section, we’d like to know a bit about you and your practice setting. Please remember that all responses are confidential and will be reported in aggregate only.

1. Have you completed a pediatric urology fellowship?
   1. Yes
   2. No
   3. Currently in a pediatric urology fellowship
2. How many years have you been in practice?
   1. Currently in fellowship
   2. 0-5
   3. 6-10
   4. 11-15
   5. 16-20
   6. >20
3. What percentage of your practice is devoted to pediatric urology?
   1. 0-24%
   2. 25-49%
   3. 50-74%
   4. 75%+
4. How many pediatric urologists are in your practice?
   1. 1-2
   2. 3-4
   3. 5-6
   4. 7-10
   5. >10
5. What is your practice setting?
   1. Academic affiliation
   2. Private practice
   3. Hospital Employee
   4. Other __________________________________
6. What is your age?
   1. <30
   2. 31-40
   3. 41-50
   4. 51-60
   5. 61+
7. What is your gender?
   1. Male
   2. Female
   3. Other (specify)_________________________________
8. What AUA section do you belong to?
   1. Mid Atlantic
   2. New England
   3. New York
   4. North Central
   5. Northeastern
   6. South Central
   7. Southeastern
   8. Western
   9. From another geographic area__________________________
9. Have you had a patient with a serious complication (requiring ICU care or an invasive procedure) or death related to a catheter-associated urinary tract infection?
   1. Yes
   2. No
   3. Don’t know
10. If yes, did you change your practice based on #9?
    1. Yes
    2. No
    3. Don’t know
    4. N/A
